# COX-2/PGE2/EP4轴诱导巨噬细胞功能活化在NSCLC发展过程中的作用

**DOI:** 10.3779/j.issn.1009-3419.2024.101.05

**Published:** 2024-04-20

**Authors:** Juan ZHAO, Qianying ZHU, Yu ZHANG, Guiyun LI, Yinglin ZHANG, Fangfang LI, Li BIAN

**Affiliations:** ^1^650032 昆明，昆明医科大学第一附属医院病理科; ^1^Department of Pathology, The First Affliated Hospital of Kunming Medical University, Kunming 650032, China; ^2^629000 遂宁，遂宁市中心医院病理科; ^2^Department of Pathology, Suining Central Hospital, Suining 629000, China; ^3^663000 文山，文山市人民医院; ^3^Wenshan People's Hospital, Wenshan 663000, China

**Keywords:** 肺肿瘤, PGE2, EP4, 巨噬细胞, 肿瘤免疫, Lung neoplasms, PGE2, EP4, Macrophage, Tumor immunity

## Abstract

**背景与目的:**

肿瘤微环境（tumor microenvironment, TME）是肿瘤发生、发展的重要因素之一，其中肿瘤相关巨噬细胞（tumor-associated macrophages, TAMs）在非小细胞肺癌（non-small cell lung cancer, NSCLC）进展中起着重要作用。然而，TAMs在NSCLC发展过程中的作用机制仍不清楚，因此本研究旨在探讨TAMs在NSCLC发展过程中的作用，并寻找潜在的治疗靶点。

**方法:**

使用GEPIA（Gene Expression Profiling Interactive Analysis）数据库分析前列腺素E2受体4（prostaglandin E2 receptor 4, EP4）mRNA在NSCLC和正常肺组织中的表达情况；利用免疫组化（immunohistochemistry, IHC）方法检测120例NSCLC组织及24例癌旁组织标本中环氧合酶-2（cyclooxygenase-2, COX-2）、EP4、分化簇86（cluster of differentiation 86, CD86）、CD163、CD31的蛋白表达水平；建立裸鼠肺腺癌细胞A549与巨噬细胞RAW264.7共移植瘤模型，使用EP4抑制剂E7046灌胃，收集样本行苏木素-伊红（hematoxylin-eosin, HE）、IHC和免疫荧光（immunofluorescence, IF）染色，免疫印迹（Western blot）检测各组裸鼠肿瘤组织上皮间充质转化（epithelial-mesenchymal transformation, EMT）相关蛋白表达情况；全长转录组测序技术筛选引起肿瘤肝转移的关键基因并进行KEGG（Kyoto Encyclopedia of Genes and Genomes）富集分析。

**结果:**

EP4 mRNA在NSCLC组织中表达水平普遍低于正常肺组织（P<0.05） ；COX-2、EP4、CD163、CD31蛋白在NSCLC组织和癌旁组织中差异性表达且在NSCLC患者多项临床病理参数中存在差异；RAW264.7缩短了A549的成瘤潜伏期，促进肿瘤增殖及肿瘤肝转移，E7046可降低裸鼠肿瘤细胞增殖活性、肿瘤组织血管密度及M2型巨噬细胞浸润；IF染色显示巨噬细胞主要分布在肿瘤转移灶周围；Western blot结果显示与A549单独移植组相比，A549与RAW264.7共移植组小鼠肿瘤组织中E-钙黏蛋白（E-cadherin）相对表达量明显降低，差异具有统计学意义（P<0.05），N-钙黏蛋白（N-cadherin）相对表达量上调，但差异无统计学意义（P>0.05）；全长转录组差异基因主要富集的通路为PI3K-AKT、MAPK信号通路。

**结论:**

在NSCLC发生发展过程中，COX-2/PGE2/EP4轴可能通过诱导巨噬细胞功能活化来促进肿瘤的进展，EP4可能是具有潜力的肿瘤免疫治疗新靶点。本研究为深入探讨NSCLC的发生发展机制提供了新的视角和思路，同时为开发新的NSCLC治疗策略提供了理论依据。

## Abstract (en)

**Background and objective:**

Tumor microenvironment (TME) is one of the important factors in tumorigenesis and progression, in which tumor-associated macrophages (TAMs) play an important role in non-small cell lung cancer (NSCLC) progression. However, the mechanism of TAMs in NSCLC progression remains unclear, so this study aimed to investigate the role of TAMs in NSCLC progression and to find potential therapeutic targets.

**Methods:**

Gene Expression Profiling Interactive Analysis (GEPIA) database was used to analyze the expression of prostaglandin E2 receptor 4 (EP4) mRNA in NSCLC and normal lung tissues; the protein expression levels of cyclooxygenase-2 (COX-2), EP4, cluster of differentiation 86 (CD86), CD163 and CD31 were detected by immunohistochemistry (IHC) in 120 NSCLC tissues and 24 paracancerous tissues specimens. The nude mouse lung adenocarcinoma cell A549 and macrophage RAW264.7 co-transplanted tumor model was established. And the samples were collected by gavage with EP4 inhibitor E7046, and then stained with hematoxylin-eosin (HE), IHC, and immunofluorescence (IF), and then detected by Western blot for the epithelial mesenchymal transformation (EMT) of the tumor tissues of the nude mice in each group. Western blot was used to detect the expressions of EMT related protiens in each group of nude mice; full-length transcriptome sequencing was used to screen the key genes causing liver metastasis and Kyoto Encyclopedia of Genes and Genomes (KEGG) enrichment analysis was performed.

**Results:**

EP4 mRNA expression level in NSCLC tissues was generally lower than that in normal lung tissues (P<0.05); COX-2, EP4, CD163, CD31 proteins were differentially expressed in NSCLC tissues and adjacent tissues, and differences were observed in many clinicopathological parameters of NSCLC patients; RAW264.7 shortened the latency period of tumorigenesis of A549 and promoted the proliferation of tumors and liver metastasis of tumors, and E7046 could reduce tumor cell proliferation activity, tumor tissue vascular density and M2-type macrophage infiltration in nude mice; IF staining showed that macrophages were mainly distributed around the metastatic foci of tumors; Western blot results showed that compared with A549 alone transplantation group, the relative expression of E-cadherin protein in tumor tissues of mice in A549 and RAW264.7 co-transplantation group was significantly decreased, and the difference was statistically significant (P<0.05), while the relative expression of N-cadherin protein was up-regulated, but the difference was not statistically significant (P>0.05); the main pathways enriched in the differential genes of the full-length transcriptome were the PI3K-AKT and MAPK signaling pathways.

**Conclusion:**

During NSCLC development, the COX-2/PGE2/EP4 axis may promote tumor progression by inducing macrophage functional activation, and EP4 may be a potential new target for tumor immunotherapy. This study provides new perspectives and ideas for in-depth exploration of the mechanisms of NSCLC development, as well as a theoretical basis for the development of new therapeutic strategies for NSCLC.

肺癌是全球癌症相关死亡最主要原因，严重危害人类健康^[1]^。非小细胞肺癌（non-small cell lung cancer, NSCLC）是最常见的肺癌类型，占所有肺癌的80%-85%，其中约75%的患者发现时已处于中晚期。虽然跨学科和综合治疗方法不断取得进展，但NSCLC转移患者的5年生存率仍然很低^[2]^。因此，寻找更有效的生物标志物以促进NSCLC免疫治疗的发展至关重要。肿瘤的生长不仅取决于肿瘤细胞的增殖，还涉及它们所处的肿瘤微环境（tumor microenvironment, TME）^[3]^。肿瘤相关巨噬细胞（tumor-associated macrophages, TAMs）构成TME的可塑性和异质性细胞群，可占某些实体肿瘤质量的50%^[4]^。它由M1和M2两种细胞亚型组成，脂多糖 （lipopolysaccharides, LPS）和干扰素-γ（interferon γ, IFN-γ）可通过经典活化途径诱导巨噬细胞向M1型巨噬细胞极化。另一方面，Th2细胞因子[如白介素4（interleukin-4, IL-4）和IL-13]可经替代活化途径诱导巨噬细胞向M2型巨噬细胞极化。生理情况下，巨噬细胞处于平衡状态，当肿瘤发生时，肿瘤细胞可激活巨噬细胞，将巨噬细胞由促炎的M1型转变为引起免疫抑制的M2型^[5]^。

前列腺素E2（prostaglandin E2, PGE2）是一种最具生物活性和研究最为广泛的前列腺素，通过4个G蛋白偶联的前列腺素E2受体（prostaglandin E2 receptors 1-4, EPs 1-4）起作用。研究^[6]^表明，PGE2会增加肿瘤的生长和侵袭，减少细胞凋亡，增加转移和血管生成，引起肿瘤免疫逃逸。此外，PGE2是TME中的重要参与者，不仅能诱导肿瘤生长，而且还能抑制免疫功能^[7]^。肿瘤细胞和基质细胞释放到TME中的PGE2与免疫细胞表面的EP4受体结合，可激活COX-2/PGE2通路在肿瘤局部形成免疫抑制环境，促进肿瘤细胞增殖、侵袭和迁移等恶性生物学行为^[8]^。本课题组前期研究发现肺腺癌细胞A549高分泌PGE2，单核巨噬细胞RAW264.7表达4型PGE2受体，其中以EP4表达量最高。可见，COX-2/PGE2/EP4轴在肿瘤的进展中发挥着重要作用，但其能否作为诱导巨噬细胞功能活化的关键因子来促进NSCLC的发展，尚不明确。本研究通过生物信息数据库分析EP4 mRNA在NSCLC患者中的表达情况，收集临床样本和建立裸鼠共移植瘤模型，进一步探讨了COX-2/PGE2/EP4轴在肺癌细胞和巨噬细胞互作关系中发挥的作用。

## 1 资料与方法

### 1.1 实验材料（细胞、动物、临床样本）

（1）小鼠肺腺癌A549细胞系购自中国科学院昆明动物研究所；小鼠巨噬细胞RAW264.7购自加拿大Applied Biological Materials（abm）公司。（2）SPF级BALB/c Nude裸鼠购自昆明医科大学实验动物学部，6周龄SPF级雌性BALB/c Nude裸鼠16只，体重18-22 g，随机分为4组，每组4只。（3）本研究共纳入NSCLC标本120例，包括肺腺癌107例，肺鳞癌13例；癌旁组织24例。所有标本均由昆明医科大学第一附属医院病理科提供。所有病例均由两位病理诊断医师确定病理诊断和组织分级，收集年龄、性别、肿瘤大小、淋巴结转移、远处转移等临床信息。所有标本均为手术切除标本。

### 1.2 方法

#### 1.2.1 生物信息库数据的收集与分析

利用GEPIA（Gene Expression Profiling Interactive Analysis）数据库及GTEx（Genotype-Tissue Expression）数据库结合TCGA（The Cancer Genome Atlas）数据对基因表达谱数据进行分析，通过对基因芯片高通量测序和二代测序记录肿瘤基因组的全方位信息及患者的临床信息。本课题使用GEPIA数据库分析NSCLC组织及正常肺组织中EP4 mRNA表达情况。

#### 1.2.2 裸鼠皮下移植瘤模型

取第三代细胞融合度达到80%-90%的A549、RAW264.7细胞，利用0.25%胰酶消化细胞，再用预冷的PBS洗2遍，最大程度弃上清。按基质胶: PBS=1:5的比例稀释，用基质胶稀释液重悬细胞，调整细胞浓度为6×10^5^个/mL。A549细胞单独或与RAW264.7细胞共移植于裸鼠皮下，将细胞充分混匀，吸取100 µL细胞悬液，缓慢注射于裸鼠腋后方皮下，可见一鼓起皮丘，注射完成后用棉签按压片刻，以防细胞漏出。隔天观察裸鼠生长状态，游标卡尺测量瘤体长、短径，计算瘤体体积[V=1/2（长径×短径^2^）]。待瘤体体积达到50-100 mm^3^时，两组任选一半随机分为实验组和对照组，实验组予以200 μL溶于0.5%甲基纤维素的150 mg/kg E7046灌胃，对照组予以等量0.5%甲基纤维素灌胃，每天给药一次，持续21 d，并测量肿瘤大小及裸鼠体重。

#### 1.2.3 苏木素-伊红（hematoxylin-eosin, HE）染色

瘤体剥离后，立即放入4% PFA固定，1周后酒精脱水、浸蜡包埋，制作厚度为4 μm的连续切片，再经脱蜡、水化、清洗后，常规HE染色，封片后镜下观察肿瘤组织病理改变。

#### 1.2.4 免疫组化（immunohistochemistry, IHC）染色

IHC采用EnVision两步法，DAB显色。所用一抗[COX-2、EP4、CD86、CD163与CD31、巨噬细胞标志物F4/80、精氨酸酶-1（arginase-1, Arg-1）、增殖细胞核抗原（proliferating cell nuclear antigen, PCNA）、CD31]及免疫组化试剂盒购自CST公司及福州迈新生物公司，操作步骤按试剂说明书进行。

#### 1.2.5 免疫荧光（immunofluorescence, IF）共染

石蜡4 μm切片，75 ^o^C烘片2 h，脱蜡水化、抗原修复同上。在组织周围使用免疫组化笔画圈，5%羊血清室温封闭2 h。滴加一抗4 ^o^C孵育过夜，切片恢复至室温，PBS洗3次。滴加二抗，室温孵育1 h。PBS洗3次。DAPI封片，激光共聚焦显微镜下观察、同一曝光条件下采图。

#### 1.2.6 全长转录组测序

与北京百迈客生物科技有限公司合作完成。

1.2.7 免疫印迹（Western blot）使用PIPA裂解液（含1% PMSF蛋白酶抑制剂、1%磷酸酶抑制剂）裂解裸鼠肿瘤组织30 min，BCA试剂盒检测蛋白浓度，10% SDS-PAGE凝胶电泳分离，转印至PVDF膜上。5%脱脂奶粉室温封闭2 h，一抗4 ^o^C冰箱孵育过夜。孵育结束后，二抗室温孵育2 h，采用增强化学发光系统（electrochemiluminescence, ECL）于显影仪中显影以检测目标条带。

### 1.3 统计学方法

应用软件SPSS 23.0和GraphPad Prism 7分析和绘图。分类数据资料描述采用频率和百分比，使用卡方检验和Fisher精确检验比较组间差异；计量资料采用均数±标准差（Mean±SD）描述数据，两组间比较采用独立样本t检验或Wilcoxon秩和检验，多组间比较采用单因素方差分析，连续监测样本采用重复测量方差分析，以P<0.05表示有统计学差异。

## 2 结果

### 2.1 PGE2受体EP4在NSCLC肿瘤组织和正常肺组织中的表达情况

根据GEPIA数据库分析结果，EP4 mRNA在NCSLC肿瘤组织和正常肺组织中差异性表达，差异具有统计学意义（P<0.05）（图1）。

**图 1:**
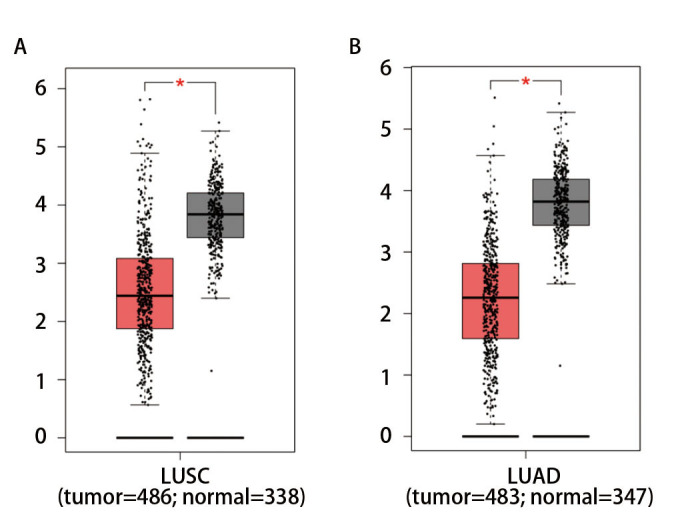
EP4 mRNA在NSCLC和正常肺组织中的表达情况。A：EP4 mRNA在LUSC和正常肺组织中的表达情况；B：EP4 mRNA在LUAD和正常肺组织中的表达情况。肿瘤组与正常组相比，*P<0.05。

### 2.2 NSCLC患者TME中COX-2、EP4、CD86、CD163、CD31的表达情况

COX-2、EP4、CD86、CD163、CD31蛋白在NSCLC和癌旁组织中的表达情况见图2。结果显示，COX-2、EP4、CD86、CD163与CD31均在NSCLC肿瘤组织和癌旁组织中差异性表达，差异具有统计学意义（P<0.05）（表1）。

**图 2:**
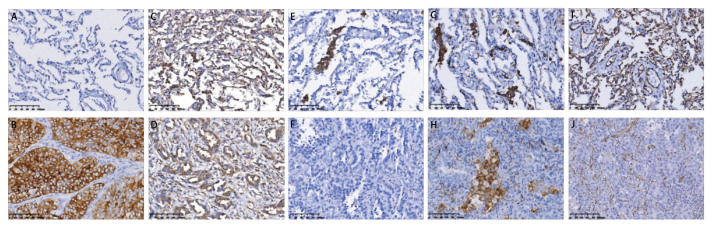
COX-2、EP4、CD86、CD163、CD31在NSCLC中的表达情况（IHC，×200）。A、B：癌旁组织（A）及NSCLC组织标本（B）COX-2 IHC染色；C、D：癌旁组织（C）及NSCLC组织标本（D）EP4 IHC染色；E、F：癌旁组织（E）及NSCLC组织标本（F）CD86 IHC染色；G、H：癌旁组织（G）及NSCLC组织标本（H）CD163 IHC染色；I、J：癌旁组织（I）及NSCLC组织标本（J）CD31 IHC染色。

**表 1:** 24例癌旁组织及120例NSCLC组织标本COX-2、EP4、CD86、CD163、CD31蛋白表达情况

Category	COX-2		χ²	P	EP4		χ²	P	CD86		χ²	P	CD163		χ²	P	CD31-MVD (pcs/mm^3^)	Z	P
	(-)	(+)			(-)	(+)			(-)	(+)			(-)	(+)					
Paraneoplastic	23	1	9.833	0.002	5	19	10.764	0.001	5	19	37.911	<0.001	1	23	6.349	0.012	8.45±4.42	˗7.700	<0.001
NSCLC	76	44			69	51			99	21			34	86			26.67±5.51		

### 2.3 COX-2、EP4、CD86、CD163、CD31蛋白在NSCLC中的表达情况及其与NSCLC临床病理特征的关系

纳入患者的临床病理参数见表2。COX-2蛋白表达在NSCLC远处转移、微乳头成分、分化程度及肿瘤原发灶-淋巴结-转移（tumor-node-metastasis, TNM）分期方面存在差异。EP4蛋白表达在NSCLC不同组织类型、肿瘤大小及分化程度方面存在差异。CD86蛋白表达在NSCLC患者年龄及远处转移方面存在差异。CD163蛋白表达在NSCLC肿瘤大小、淋巴结转移及分化程度方面存在差异。CD31蛋白表达强度在NSCLC不同组织类型、肿瘤大小、淋巴结转移、远处转移、分化程度及TNM分期方面存在差异，以上差异均具有统计学意义（P<0.05）（表2）。

**表 2:** NSCLC患者临床病理特征及其与COX-2、EP4、CD86、CD163、CD31蛋白表达情况的单因素分析

Category	n (%)	COX-2		χ²	P	EP4		χ²	P	CD86		χ²	P	CD163		χ²	P	CD31-MVD (pcs/mm^3^)	t/F	P
		(-)	(+)			(-)	(+)			(-)	(+)			(-)	(+)					
Age (yr)				0.293	0.588			1.841	0.175			5.089	0.024			0.027	0.869		0.925	0.357
≤60	72 (60.0)	47	25			45	27			64	8			20	52			27.05±5.62		
>60	48 (40.0)	29	19			24	24			35	13			14	34			26.10±5.33		
Tumor diameter				0.218	0.641			13.777	<0.001			0.174	0.677			11.120	0.001		-3.549	0.001
≤3 cm	87 (72.5)	54	33			59	28			71	16			32	55			25.62±5.13		
>3 cm	33 (27.5)	22	11			10	23			28	5			2	31			29.44±5.59		
Tissue type				0.026	0.871			7.069	0.008			0.030	0.862			2.025	0.155		-2.314	0.022
Adenocarcinoma	107 (89.2)	67	40			66	41			89	18			33	74			26.27±5.37		
Squamous carcinoma	13 (10.8)	9	4			3	10			10	3			1	12			29.95±5.72		
Lymph node metastasis				2.123	0.145			2.604	0.107			3.534	0.060			9.856	0.002		˗14.877	<0.001
No	89 (74.2)	53	36			55	34			70	19			32	57			24.24±3.94		
Yes	31 (25.8)	23	8			14	17			29	2			2	29			33.65±2.64		
Distant metastasis				6.605	0.010			1.612	0.204			4.195	0.041			0.233	0.629		-3.785	<0.001
No	85 (70.8)	60	25			52	33			74	11			23	62			25.52±5.23		
Yes	35 (29.2)	16	19			17	18			25	10			11	24			29.49±5.20		
Micronipple				21.958	<0.001			0.158	0.691			1.653	0.199			0.484	0.487		-0.543	0.588
No	61 (50.8)	51	10			34	27			53	8			19	42			26.40±5.35		
Yes	59 (49.2)	25	34			35	24			46	13			15	44			26.95±5.70		
Differentiation degree				11.953	0.003			9.507	0.009			0.467	0.792			9.268	0.010		15.026	<0.001
High	33 (27.5)	29	4			26	7			26	7			16	17			23.15±4.98		
Middle	54 (45.0)	30	24			29	25			45	9			12	42			26.91±5.24		
Low	33 (27.5)	17	16			14	19			28	5			6	27			29.81±4.41		
TNM stage				18.147	<0.001			6.180	0.103			1.131	0.770			6.587	0.086		25.205	<0.001
I	53 (44.2)	38	15			37	16			44	9			19	34			22.91±3.27		
II	29 (24.2)	23	6			13	16			24	5			3	26			30.06±4.48		
III	23 (19.1)	12	11			12	11			20	3			8	15			28.36±4.59		
IV	15 (12.5)	3	12			7	8			11	4			4	11			29.27±6.55		

### 2.4 巨噬细胞促进肿瘤生成与增殖且E7046可减少肿瘤组织中M2型巨噬细胞的浸润并降低肿瘤增长速度和血管密度

通过肺腺癌细胞A549和巨噬细胞RAW264.7裸鼠荷瘤模型发现，RAW264.7缩短了A549的成瘤时间，加快了肿瘤增长速度。E7046减缓了肿瘤生长速度，但对小鼠体重无明显影响（图3A、3B）。HE染色结果显示，与A549单独移植相比，A549与RAW264.7共移植小鼠肿瘤组织中坏死成分较多，使用E7046后，共移植小鼠肿瘤坏死成分相对减少（图3C）。IHC结果发现A549与RAW264.7共移植后，肿瘤组织中F4/80、Arg-1蛋白表达量显著上调，E7046可降低小鼠肿瘤组织中F4/80、Arg-1的表达（图4A、4B）。共移植后肿瘤组织中PCNA、CD31蛋白表达量增加，E7046可降低PCNA、CD31的表达（图4C、4D）。

**图 3:**
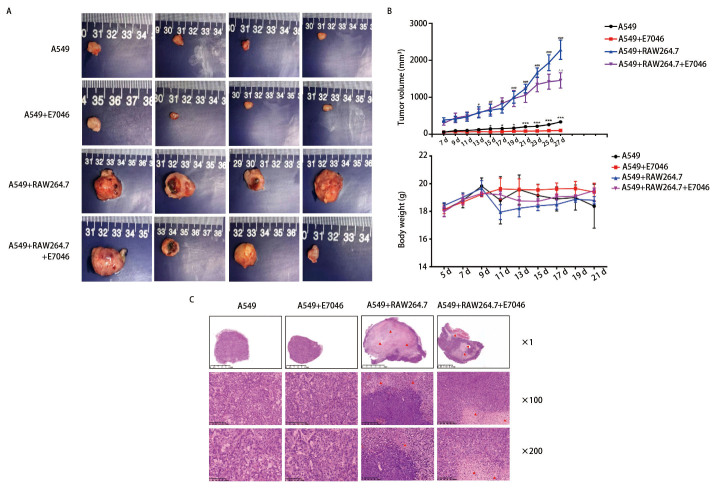
裸鼠肺癌细胞与巨噬细胞共移植模型的构建。A、B：裸鼠皮下A549与RAW264.7移植瘤模型及裸鼠肿瘤体积及体重统计图。A549组与A549+E7046组相比，^*^P<0.05，^***^P<0.001；A549组与A549+RAW264.7组相比，^#^P<0.05，^##^P<0.01，P<0.001；A549+RAW264.7组与A549+RAW264.7+E7046组相比，^ʌʌ^P<0.01；C：裸鼠皮下移植瘤HE染色。

**图 4:**
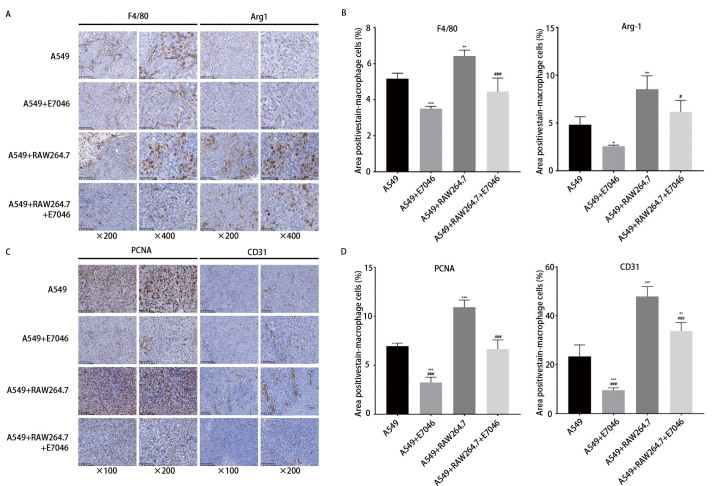
裸鼠共移植模型中巨噬细胞对肺癌细胞增殖的影响。A、B：裸鼠皮下移植瘤模型巨噬细胞标志物F4/80、Arg-1 IHC染色；C、D：裸鼠皮下移植瘤模型PCNA、CD31 IHC染色。各组与A549组相比，*P<0.05，**P<0.01，***P<0.001；A549+RAW264.7+E7046组与A549+RAW264.7组相比，#P<0.05，P<0.001。

### 2.5 巨噬细胞促进肺癌细胞肝转移且E7046可降低转移结节数且延缓EMT进程

我们发现，在A549与RAW264.7共移植小鼠身上均出现肿瘤肝转移，对小鼠肝脏组织进行了Pan-keratin和F4/80免疫荧光双色共染，发现肿瘤转移灶表达角蛋白、F4/80阳性的巨噬细胞主要分布在肿瘤转移灶周围。给予E7046后肝脏转移结节数减少，F4/80阳性的巨噬细胞数量也较对照组减少（图5A-5C）。但我们未检测到肝脏转移结节数差异有统计学意义，可能与给药时间、观察时间较短及药剂用量较低有关。Western blot结果显示，与A549单独移植组相比，A549与RAW264.7共移植组小鼠肿瘤组织中E-cadherin蛋白相对表达量明显降低，差异具有统计学意义（P<0.05），单独移植组和共移植组小鼠予以E7046处理后，肿瘤组织中E-cadherin蛋白相对表达量均上调，差异无统计学意义（P>0.05）（图5D）。与A549单独移植组相比，共移植组小鼠肿瘤组织中N-cadherin蛋白相对表达量上调，但差异无统计学意义（P>0.05），A549单独移植组小鼠经E7046处理后，肿瘤组织中N-cadherin蛋白相对表达量明显降低，差异具有统计学意义（P<0.001），A549与RAW264.7共移植组小鼠经E7046处理后，肿瘤组织中N-cadherin蛋白相对表达量降低，差异无统计学意义（P>0.05）（图5E）。

**图 5:**
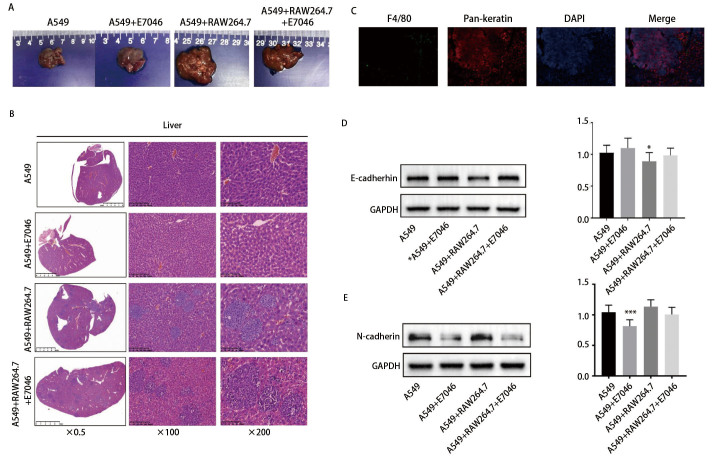
小鼠肝脏组织学染色及EMT相关蛋白表达情况。A：裸鼠肝脏大体图；B：裸鼠肝脏HE染色；C：A549+RAW264.7组裸鼠肝脏Pan-keratin与F4/80 IF双色共染；D：蛋白印迹检测小鼠肿瘤组织E-cadherin的表达情况；E：Western blot检测小鼠肿瘤组织N-cadherin的表达情况。与A549组相比，*P<0.05，***P<0.001。

### 2.6 差异表达基因通路富集分析

为探究引起共移植小鼠肺癌细胞肝转移可能的差异基因，对单独移植组、共移植组肿瘤组织及共移植组裸鼠肝脏组织进行全长转录组测序。结果显示A549单独移植组与A549与RAW264.7共移植组肿瘤组织共鉴定出差异表达基因24,480个，其中8951个基因表达上调；15,529个基因表达下调（图6A）。单独移植组肿瘤组织与共移植组肝脏组织共鉴定出差异表达基因26,561个，其中10,073个基因表达上调，16,488个基因表达下调（图6B）。对样本肿瘤组织与肝组织前20个差异表达基因进行分析，结果发现前20个差异表达基因中有16个基因在两组间均表达，其中C15orf40、TFPI2、AC253536.7、SLC22A18AS、C11orf71、ONT.5312基因表达量差异倍数最大（图6C、6D）。进行KEGG（Kyoto Encyclopedia of Genes and Genomes）富集分析发现，在单独移植组和共移植组肿瘤组织中，差异基因主要富集在阿尔兹海默症（Alzheimer disease）、肌萎缩性侧束硬化症（Amyotrophic lateral sclerosis）、亨廷顿病（Huntington disease）等（图7A）。在单独移植组肿瘤组织与共移植组肝组织样本中，差异基因主要富集在亨廷顿病、帕金森症（Parkinson disease）、朊病毒病（Prion disease）等（图7B）。对差异表达基因KEGG的注释结果按照KEGG中通路类型进行分类。结果显示与单独移植组肿瘤组织相比，共移植组肿瘤组织及肝组织样本差异表达基因均在癌症通路中最为集中，分别涉及743个和258个差异表达基因，在信号通路方面PI3K-AKT信号通路、MAPK通路均有较多的差异基因富集（图7C、7D）。

**图 6:**
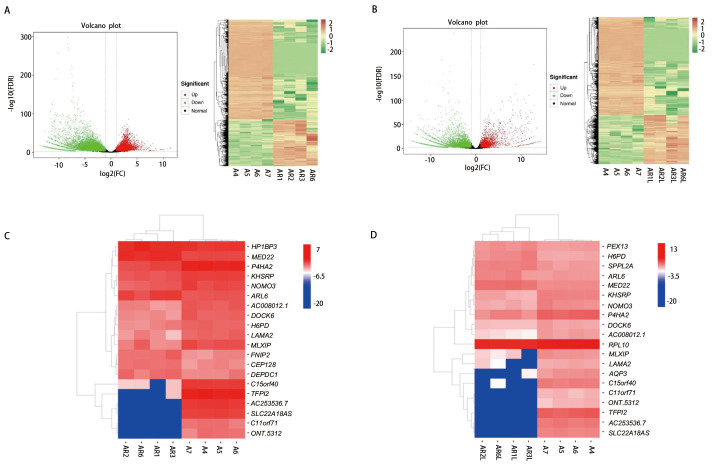
全长转录组测序技术筛选引起肺癌肝转移的关键基因。A、B：A组与AR组及A组与ARL组差异表达基因火山图和热图。绿色代表下调差异表达基因，红色代表上调差异表达基因；横坐标绝对值越大两样品间表达量差异倍数越大；纵坐标值越大差异表达越显著；C、D：A组与AR组及A组与ARL组前20个差异表达基因热图。A组：A549组肿瘤组织，AR组：A549+RAW264.7组肿瘤组织，ARL组：A549+RAW264.7组肝组织。

**图 7:**
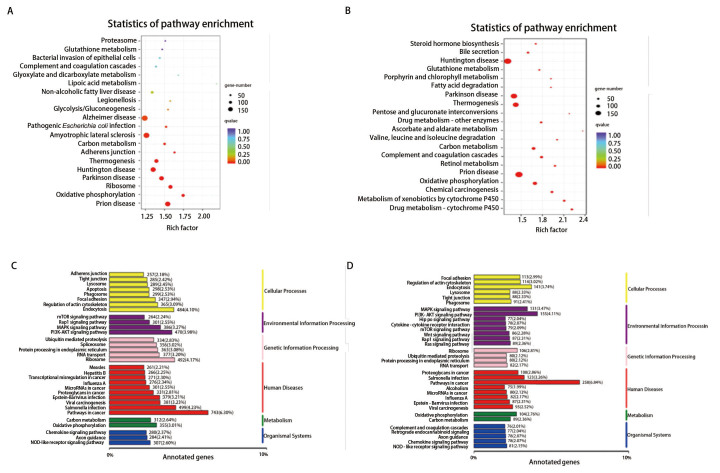
差异表达基因KEGG富集通路分析。A、B：A组与AR组及A组与ARL组KEGG通路富集散点图（每一个圆表示一个KEGG通路，纵坐标表示通路名称，横坐标为富集因子。富集因子越大，表示差异表达基因在该通路中的富集水平越显著。圆圈的颜色代表q value；大小表示通路中富集的基因数目，圆圈越大基因越多）；C、D：A组与AR组及A组与ARL组差异表达基因KEGG分类图（纵坐标为KEGG代谢通路的名称，横坐标为注释到该通路下的基因个数及其个数占被注释上的基因总数的比例）。其中A4、A5、A6、A7为A549组裸鼠皮下移植瘤组织样本；AR1、AR2、AR3、AR6为A549+RAW254.7组裸鼠皮下移植瘤组织样本；AR1L、AR2L、AR3L、AR6L为A549+RAW254.7组裸鼠肝组织样本。

## 3 讨论

肺癌是全球癌症相关死亡的主要原因，每年估计有200万新增病例和176万死亡病例^[1]^。NSCLC是肺恶性肿瘤的主要病理类型之一，其发病率及死亡率高，晚期NSCLC患者的5年生存率不超过5%^[9,10]^，因此迫切需要寻找可靠的生物标志物为NSCLC患者提供更早更明确的诊断以及更精准的靶向治疗。PGE2由花生四烯酸通过酶COX-2生成，在炎症环境及TME中起着重要作用，是TME中的重要参与者。PGE2参与调节多个免疫应答细胞的功能，包括T淋巴细胞、自然杀伤细胞、树突状细胞、巨噬细胞和髓样来源的抑制细胞^[11]^。有研究^[12]^报道，肿瘤细胞在PGE2刺激后分泌的成纤维细胞生长因子1（fibroblast growth factor 1, FGF1）可以促进肿瘤相关成纤维细胞（tumor-associated fibroblasts, CAFs）增殖和血管内皮生长因子A（vascular endothelial growth factor A, VEGFA）表达。EP4是PGE2调控肿瘤细胞恶性生物学行为的主要参与者，主要表达于髓样细胞和T淋巴细胞，可以抑制固有免疫和适应性抗肿瘤免疫应答^[13,14]^。既往对于肿瘤发展过程中COX-2/PGE2/EP4信号通路作用的研究主要集中于肿瘤细胞本身，随着近年来对肿瘤的深入研究^[15,16]^，人们发现COX-2/PGE2/EP4信号通路也可通过调节骨髓来源抑制细胞、TAMs、T细胞、自然杀伤细胞等免疫细胞调控肿瘤免疫微环境，使肿瘤细胞发生免疫逃逸，抑制机体抗肿瘤免疫反应。深入探究COX-2/PGE2/EP4轴在肺癌TME诱导巨噬细胞活化中的作用，有助于寻找新的潜在的免疫治疗靶点，为NSCLC诊治提供新思路。

本研究在GEPIA数据库分析基础上，收集临床样本，采用IHC检测NSCLC组织及癌旁组织中COX-2、EP4、CD86、CD163、CD31的蛋白表达情况并分析其与临床参数的关系。发现EP4 mRNA在肺癌和正常肺组织中差异性表达。IHC结果显示EP4蛋白在癌旁组织中的阳性表达率高于NSCLC组织，与GEPIA数据库分析结果一致，而EP4上游的COX-2蛋白在NSCLC组织中的阳性表达率高于癌旁组织，巨噬细胞标志物CD86、CD163蛋白在癌旁组织中的阳性表达率高于NSCLC组织，血管标志物CD31在NSCLC组织高于癌旁组织。COX-2蛋白表达在NSCLC远处转移、微乳头成分、分化程度及TNM分期方面存在差异。EP4蛋白表达在NSCLC不同组织类型、肿瘤大小及分化程度方面存在差异。CD86蛋白表达在NSCLC患者年龄及远处转移方面存在差异。CD163蛋白表达在NSCLC肿瘤大小、淋巴结转移及分化程度方面存在差异。CD31蛋白表达强度在NSCLC不同组织类型、肿瘤大小、淋巴结转移、远处转移、分化程度及TNM分期方面存在差异，差异均具有统计学意义。以上结果证实COX-2、EP4、CD163、CD31蛋白在NSCLC组织和癌旁组织中差异性表达且在NSCLC患者多项临床病理参数中存在差异，初步探索了COX-2/PGE2/EP4轴可能参与调控了巨噬细胞的极化以及其在肿瘤进展中发挥的重要作用。

有研究^[17]^发现人间充质干细胞产生的PGE2可将巨噬细胞从促炎的M1表型转变为抗炎的M2表型。在小鼠胃癌模型中幽门螺旋杆菌和PGE2可上调细胞因子趋化因子配体2（cytokine chemokine ligand 2, CCL2），募集巨噬细胞至肿瘤部位激活Wnt信号通路，促进胃癌的发生^[18]^。抑制EP4可降低小鼠CT26结肠癌模型中M2型巨噬细胞的表达，并增强肿瘤组织中细胞毒性T淋巴细胞的浸润，抑制肿瘤的生长^[19]^。有研究^[13]^也发现在结直肠癌（colorectal cancer, CRC）中敲除或使用药物抑制EP4受体可诱导巨噬细胞向抗肿瘤的M1表型极化。我们建立了裸鼠皮下肺腺癌细胞A549与巨噬细胞RAW264.7共移植瘤模型，研究TME中巨噬细胞与肺癌细胞的互作关系，并采用受体阻断技术，进一步探究PGE2/EP4轴作为肿瘤免疫治疗靶点的可行性和具体作用机制。结果发现巨噬细胞缩短了小鼠的成瘤潜伏期，加快肿瘤增殖速度，增加肿瘤血管密度和M2型巨噬细胞的浸润，促进肿瘤肝转移，E7046可部分逆转上述现象，降低转移结节数且延缓上皮间充质转化（epithelial-mesenchymal transformation, EMT）进程。证实了在肺癌TME中M2型-TAMs可促进肿瘤生长并增加肿瘤血管密度，同时为以PGE2/EP4信号通路为靶点的治疗提供了理论依据。

为探究小鼠移植瘤模型中出现肺癌肝转移的关键基因，我们进行了肿瘤组织和肝组织全长转录组测序。对A549单独移植组和A549与RAW264.7共移植组肿瘤组织及共移植组肝组织前20个差异表达基因进行分析，前20个差异表达基因中有16个基因在两组间均表达，以C15orf40、TFPI2、AC253536.7、SLC22A18AS、C11orf71、ONT.5312基因表达量差异倍数最大，其中TFPI2已报道和多种肿瘤相关，在乳腺癌组织和细胞系中TFPI2表达下调，与患者预后不良相关^[20]^，TFPI2还可以抑制黑色素瘤的血管性表型^[21]^。但也有文献^[22]^报道显示TFP12在肿瘤中发挥着相反的作用，如CRC中TFPI2被高甲基化，提示CRC的不良预后，PARP1也可通过TFPI2依赖性方式促进血管平滑肌细胞（vascular smooth muscle cells, VSMC）的增殖和迁移，从而加速了高血糖诱导的新生内膜增生过程^[23]^。以上研究均表明TFP12与多种肿瘤进展密切相关，但在NSCLC中尚未报道，结合本研究测序结果，可进一步探讨其在NSCLC与巨噬细胞互作关系中发挥的作用。通过KEGG富集分析我们发现参与氧化磷酸化、PI3K/AKT信号通路调控的差异基因中可能存在巨噬细胞促进肿瘤肝转移的关键基因。包括巨噬细胞在内的肿瘤基质细胞和癌细胞之间的相互代谢作用在肿瘤免疫逃避和癌症干细胞持续存在中发挥着重要意义^[24]^。促肿瘤的M2型巨噬细胞可优先使用氧化磷酸化来满足其代谢需求，而抗肿瘤的M1型巨噬细胞则使用糖酵解作为其主要代谢来源，代谢系统的失调是巨噬细胞从M1向M2表型状态倾斜的驱动力^[25]^。我们推测氧化磷酸化信号通路相关差异基因可能影响肿瘤细胞生长、运动、增殖过程以促进肿瘤细胞转移，具体差异基因及机制仍然需要进一步研究，以期为NSCLC的免疫靶向治疗提供新思路。
